# Supplementary material for: Propylene Production via Oxidative Dehydrogenation of Propane with Carbon Dioxide over Composite MxOy-TiO2 Catalysts
Source: Nanomaterials (Basel). 2023 Dec 28;14(1):86. doi: 10.3390/nano14010086 (PMC10780612; doi:10.3390/nano14010086)
Supplement: Supplementary file 1 [file nanomaterials-14-00086-s001.zip › nanomaterials-2763430-supplementary.pdf]

SUPPLEMENTARY MATERIAL

Propylene Production via Oxidative Dehydrogenation of Propane with Carbon Dioxide over Composite M_xO_y-TiO_2 Catalysts

Alexandra Florou [1], Georgios Bampos [2], Panagiota D. Natsi [2], Aliki Kokka [1] and Paraskevi Panagiotopoulou [1,*]

[1] Laboratory of Environmental Catalysis, School of Chemical and Environmental Engineering, Technical University of Crete, GR-73100 Chania, Greece; aflorou@tuc.gr (A.F.); akokka@tuc.gr (A.K.)
[2] Department of Chemical Engineering, University of Patras, GR-26504 Patras, Greece; geoba@chemeng.upatras.gr (G.B.); natsi@chemeng.upatras.gr (P.D.N.)
* Correspondence: ppanagiotopoulou@tuc.gr; Tel.: +30-28210-37770

Table S1. Comparison of literature results for the reaction of the CO_2-assisted ODP.

Catalyst	CO_2-assisted ODP reaction conditions (WGHSV; CO_2:C_3H_8 ratio)	T (°C)	X_{C3H8} (%)	S_{C3H6} (%)	Y_{C3H6} (%)	Ref.
Ga_2O_3-TiO_2	WGHSV= 6000 mL h^{-1} g^{-1} CO_2:C_3H_8 =5:1	700	45	36.2	16.3	This work
Gr_2O_3-TiO_2			45.6	35	16.0	
Ga_2O_3-TiO_2	WGHSV= 6000 mL h^{-1} g^{-1} CO_2:C_3H_8 =2:1	600	32	73	23.4	[15]
Ga_2O_3-Al_2O_3			26	94	24.4	
Ga_2O_3-ZrO_2			30	65	19.5	
Ga_2O_3-SiO_2			6.4	92	5.9	
Ga_2O_3-MgO			4.3	29	1.2	
Ga_2O_3-Al_2O_3	WGHSV= 6000 mL h^{-1} g^{-1} CO_2:C_3H_8 =3:1	550	35.2	95	33.4	[71]
Ga_2O_3-Al_2O_3	WGHSV= 4500 mL h^{-1} g^{-1} CO_2:C_3H_8 =5:1	600	36	82.1	29.6	[29]
Ga_2O_3-Al_2O_3	WGHSV= 3000 mL h^{-1} g^{-1} CO_2:C_3H_8 =2:1	500	49.7	91.7	45.6	[75]

CrO_x/CNTs [a]	WGHSV= 3600 mL h^{-1} g^{-1} CO_2:C_3H_8 =2:1	600	13	61.5	8.0	[73]
CrO_x/GNFs [b]			21	56.2	11.8	
CrO_x/silicalite-1	WGHSV= 3000 mL h^{-1} g^{-1} CO_2:C_3H_8 =5:1	550	50	67	33.5	[74]
Ga_2O_3-m [c]	WGHSV= 9000 mL h^{-1} g^{-1} CO_2:C_3H_8 =5:1	550	19.0	91.6	17.4	[5]
Ga_2O_3-Al_2O_3			13.2	95.8	12.6	
Ga/Na-ZSM5	WGHSV= 7200 mL h^{-1} g^{-1} CO_2:C_3H_8 =1:1	600	59	44	26.0	[27]
Ga/Na-SSZ-39			38	63	23.9	
5%CrO_x/MCM-41	WGHSV= 1800 mL h^{-1} g^{-1} CO_2:C_3H_8 =2:1	650	21	81	17.0	[8]
5%Cr/$Ce_{0.1}Zr_{0.9}O_2$	WGHSV= 6000 mL h^{-1} g^{-1} CO_2:C_3H_8 =2:1	600	75	66.7	50.1	[105]
5%Cr/ZrO_2			79.8	57.6	45.9	
5%Cr/CeO_2			20.8	79.8	16.6	

[a]: CNTs: Carbon nanotubes

[b]: GNFs: Carbon nanoflakes

[c]: m: mesoporous

Thermogravimetric analysis (TGA)

The surface acidity was defined as the micromoles of acid sites per gram of catalyst and calculated by the weight loss obtained from the TGA curves using the following equation (S1) [88].

$$Acidity = \left(\frac{\%Weight\ loss}{100}\right) \cdot \left(\frac{1}{MW_{NH3}}\right) \cdot 10^{6} \qquad [\frac{\mu mol}{g}] \qquad \text{(S1)}$$

Where MW_{NH3} is the molecular weight of ammonia (=17.031 g/mol) and *%Weight loss* is the weight loss obtained from the TGA curve in each weight loss region as indicated with the double arrows in Figure S3. Therefore, we estimated the density of weak/moderate and strong acid sites for the investigated catalysts and results obtained are shown in Table S2.

Table S2: Acid site density estimated from TGA experiments over TiO_2, 10%Cr_2O_3-TiO_2 and 10%Ga_2O_3-TiO_2

Catalyst	Acid Site Density (μmol/g)		
	**Weak/Moderate**	**Strong**	**Total**
TiO_2	139.2	170.9	310.1
10%Cr_2O_3-TiO_2	169.1	149.7	318.8
10%Ga_2O_3-TiO_2	267.2	243.1	510.3

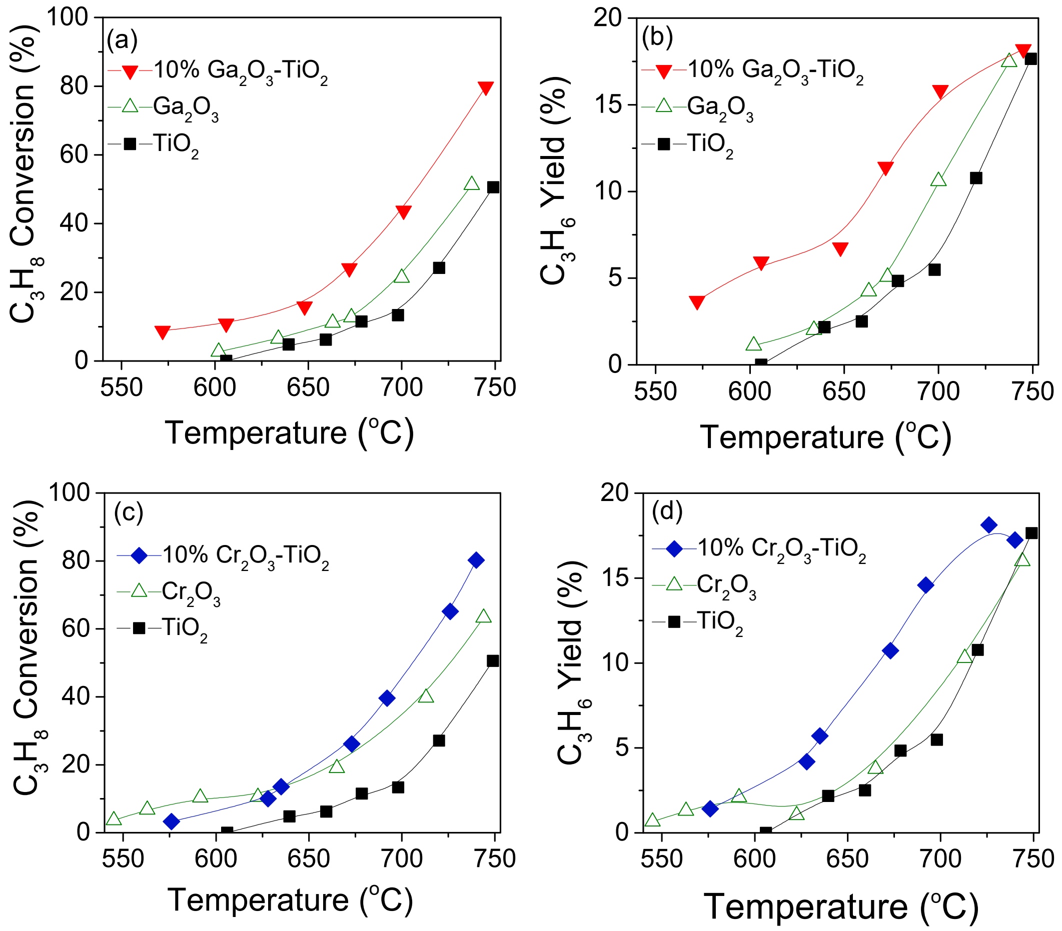

Figure S1. (a, c) Conversions of C_3H_8 and (b, d) yields of C_3H_6 as a function of reaction temperature obtained over TiO_2, Ga_2O_3, Cr_2O_3 and 10%M_xO_y-TiO_2 (M: Ga, Cr) catalysts. Experimental conditions same as in Figure 4.

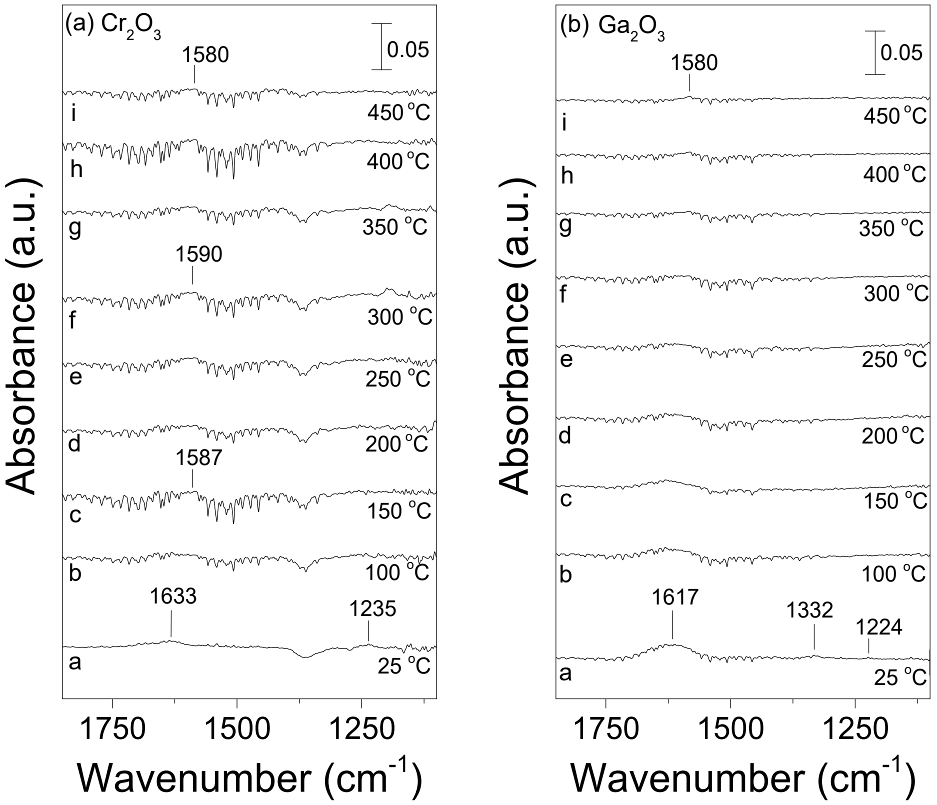

Figure S2. DRIFT spectra obtained from (a) Cr_2O_3 and (b) Ga_2O_3 catalysts following adsorption of CO_2 at 25 °C for 30 min and subsequent stepwise heating at the indicated temperatures under He flow.

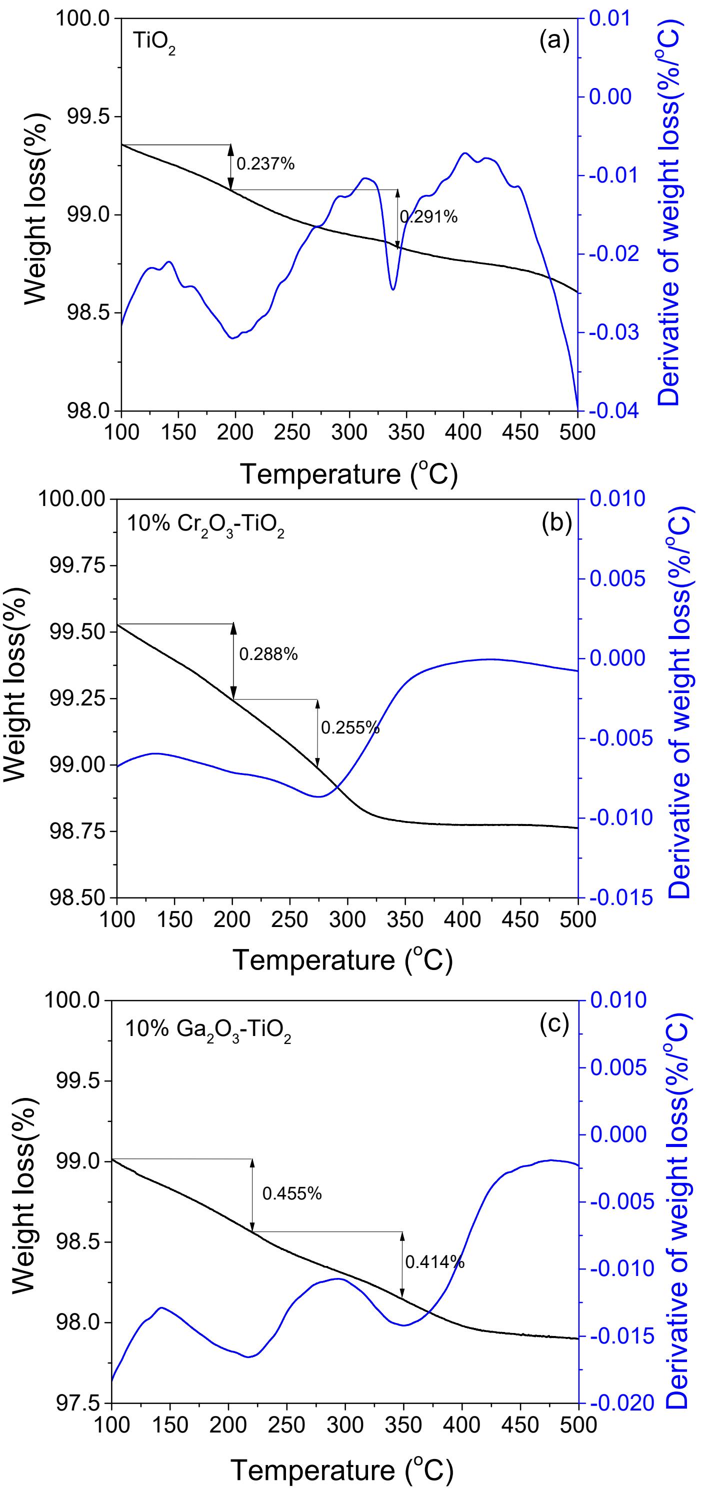

Figure S3. TGA derivative curves as a function of temperature obtained from (a) TiO_2, (b) 10%Cr_2O_3-TiO_2 and (c) 10% Ga_2O_3-TiO_2 catalysts.

15. References

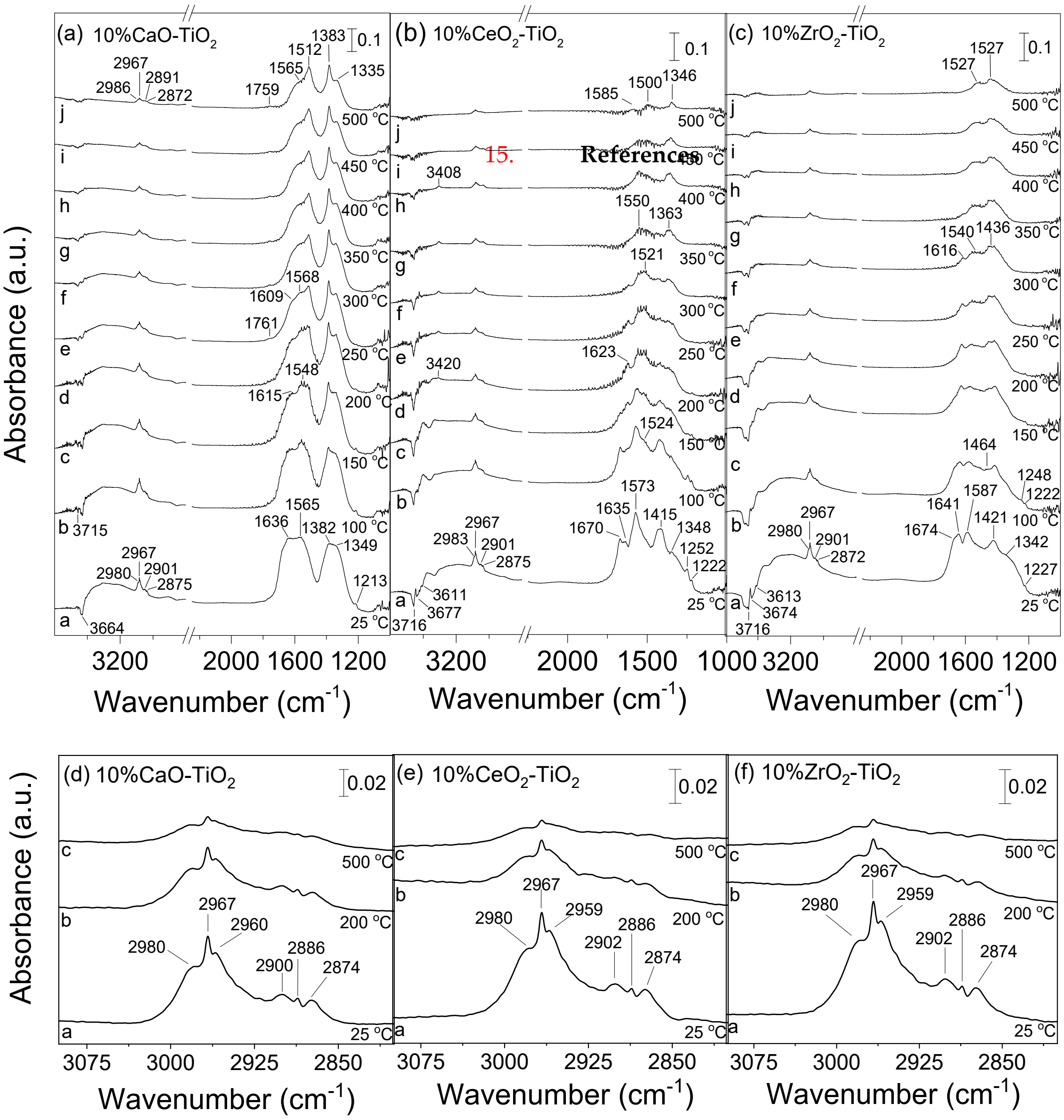

Figure S4. DRIFT spectra obtained over (a) CaO-TiO_2, (b) CeO_2-TiO_2 and (c) ZrO_2-TiO_2 catalysts following interaction with 1% C_3H_8 + 5% CO_2 (in He) at 25 °C for 15 min and subsequent stepwise heating at 500 °C. The corresponding DRIFT spectra obtained in the 3100–2750 cm^{-1} region are presented in (d), (e) and (f).
